# Altered expression of target genes of spinal cord in different itch models compared with capsaicin assessed by RT-qPCR validation

**DOI:** 10.18632/oncotarget.20148

**Published:** 2017-08-10

**Authors:** Bao-Wen Liu, Zhi-Xiao Li, Zhi-Gang He, Cheng Liu, Jun Xiong, Hong-Bing Xiang

**Affiliations:** ^1^ Department of Anesthesiology and Pain Medicine, Tongji Hospital, Tongji Medical College, Huazhong University of Science and Technology, Wuhan, China; ^2^ Hepatobiliary Surgery Center, Union Hospital, Tongji Medical College, Huazhong University of Science and Technology, Wuhan, China

**Keywords:** itch, spinal cord, capsaicin, microarray, RT-qPCR

## Abstract

Spinal cord plays a central role in the development and progression of pathogenesis of obstinate pruritus. In the current study, four groups of adult male C57Bl/6 mice were investigated; one group treated with saline, while the other groups intradermally injected with compound 48/80, histamine, α-Me-5-HT and capsaicin (algogenic substance), respectively. The intradermal microinjection of pruritic and algogenic compound resulted in a dramatic increase in the itch/algogenic behavior. Analysis of the microarray data showed that 15 genes in spinal cord (C5-C8) were differentially expressed between control group and 48/80 group, in which 9 genes were up-regulated and 6 genes were down-regulated. Furthermore, the results of RT-qPCR validation studies in C5-C8 spinal cord revealed that the 9 mRNA (Sgk1, Bag4, Fos, Ehd2, Edn3, Wdfy, Corin, 4921511E18Rik and 4930423020Rik) showed very different patterns for these different drugs, especially when comparing α-Me-5-HT and capsaicin. In three itch models, Fos and Ehd2 were up-regulated whereas Corin, 4921511E18Rik and 4930423020Rik were down-regulated. Furthermore, Corin and 4930423020Rik were down-regulated in itch model group compared to capsaicin group. Thus the application of microarray technique, coupled with RT-qPCR validation, further explain the mechanism behind itching evoked by pruritic compounds. It can contribute to our understanding of pharmacological methods for prevention or treatment of obstinate pruritus.

## INTRODUCTION

The majority of humans have experienced mild to moderate pruritus. Mosquito biting evoked-acute itching is one of the most common types of moderate pruritus. Some patients with serious skin diseases such as atopic dermatitis, advanced liver diseases or renal diseases, will experience moderate to severe itching. Obstinate pruritus is a pathological condition that affects skin sensory processing [1–4]. The mechanisms responsible for intractable pruritus are poorly understood, but refractory itching seems to be enhanced by a state of spinal hypersensitivity. It was reported that the spinothalamic tract implicated the spinal cord as a critical site for scratch-evoked suppression of itch [5–8]. Study of Sun et al has indicated that the gastrin-releasing peptide receptor (GRPR) in the dorsal horn of the spinal cord represent the first molecule that is dedicated to mediating the itch sensation [9]. It is well-known that GRPR-positive neurons in the spinal cord constitute a long-sought labeled line for itch not pain sensation [10]. In our previous research, the results of proton nuclear magnetic resonance (^1^H-NMR) studies of the spinal cord extracts revealed that the metabolites showed very different patterns for pruritogen and algogenic substance [11]. Given the high prevalence of itch in some patients with skin diseases [12–14], it is pivotal to know whether the genes are dysregulated in the spinal cord in different itch models.

Previous studies from pain data indicate that identification of whole genome messenger RNA (mRNA) expression profiles provide insight into the widespread factors that contribute to the development of more effective treatments for intractable pain [15–17]. Analysis using expression microarrays has revealed that many genes are regulated following noxious stimuli such as pruritus. In the present study, whole genome mRNA expression profiles in spinal cords of mice with compound 48/80 evoked-itching were analyzed to identify as unique target genes. Further, these candidate genes were examined in different itch models so as to provide central therapeutic targets for antipruritic drug development.

## RESULTS

### mRNA expression profiling revealed itch-related genes in lower cervical spinal cord from compound 48/80-evoked pruritus mice

To identify altered genes that might contribute to spinal hypersensitivity of acute itch, we conducted itch mRNA profiling experiment on mouse acute itching models by compound 48/80. The mRNA expressions of lower cervical spinal cord (C5-C8) of the animals were examined using Affymetrix Mouse Genome 430 2.0 microarrays that include 10,000 probe sets. The gene expression profiles in the 48/80 group were compared with the corresponding data of control group. We identified that a total of 15 probe sets were differentially expressed between control group and 48/80 group by the microarray data analysis, in which 9 probe sets were up-regulated and 6 probe sets were down-regulated (Figure 2). The maximal and minimal fold change was 5.16 and 2.01, respectively.

**Figure 1:**
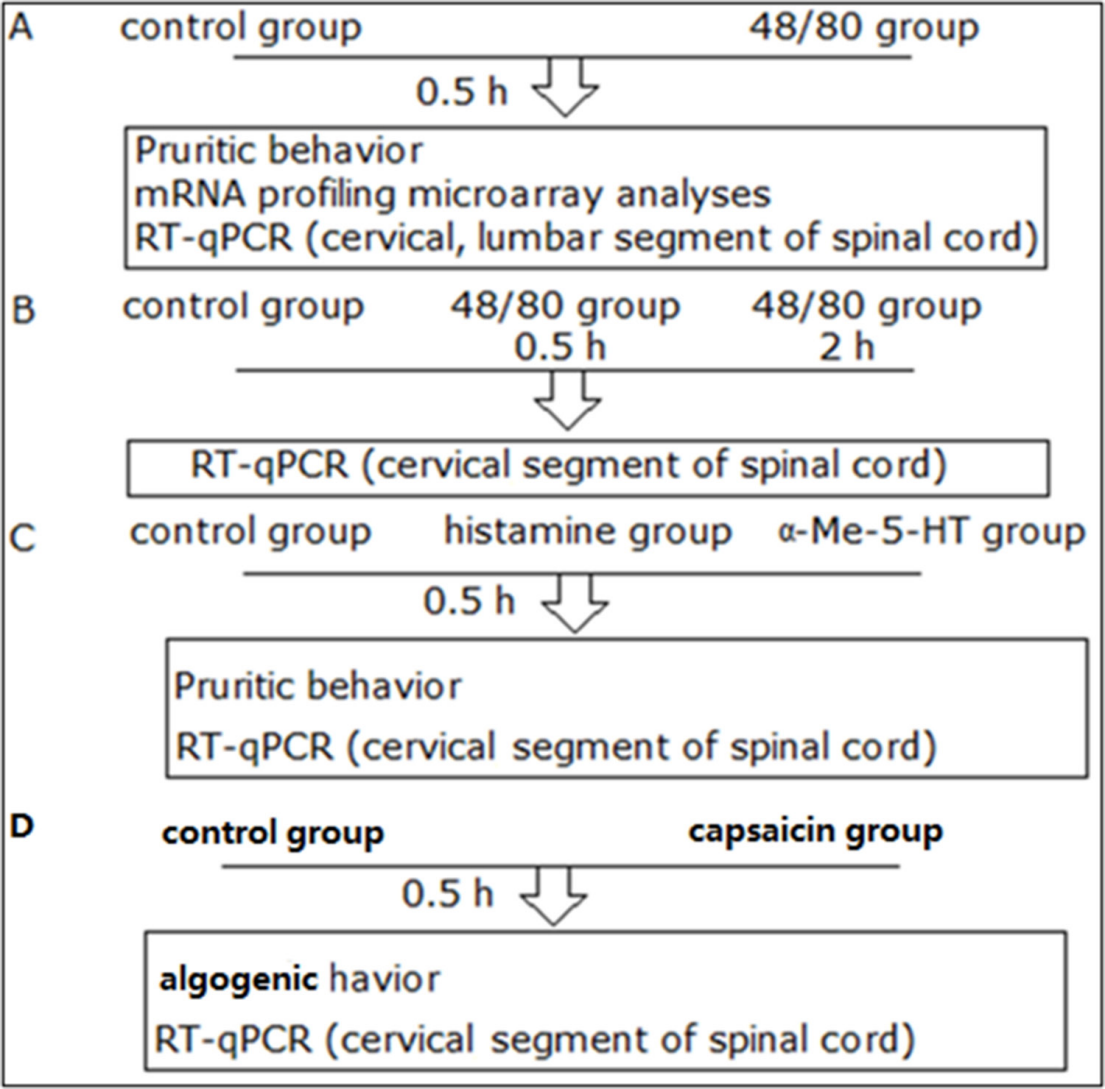
An illustration of the experimental design

**Figure 2:**
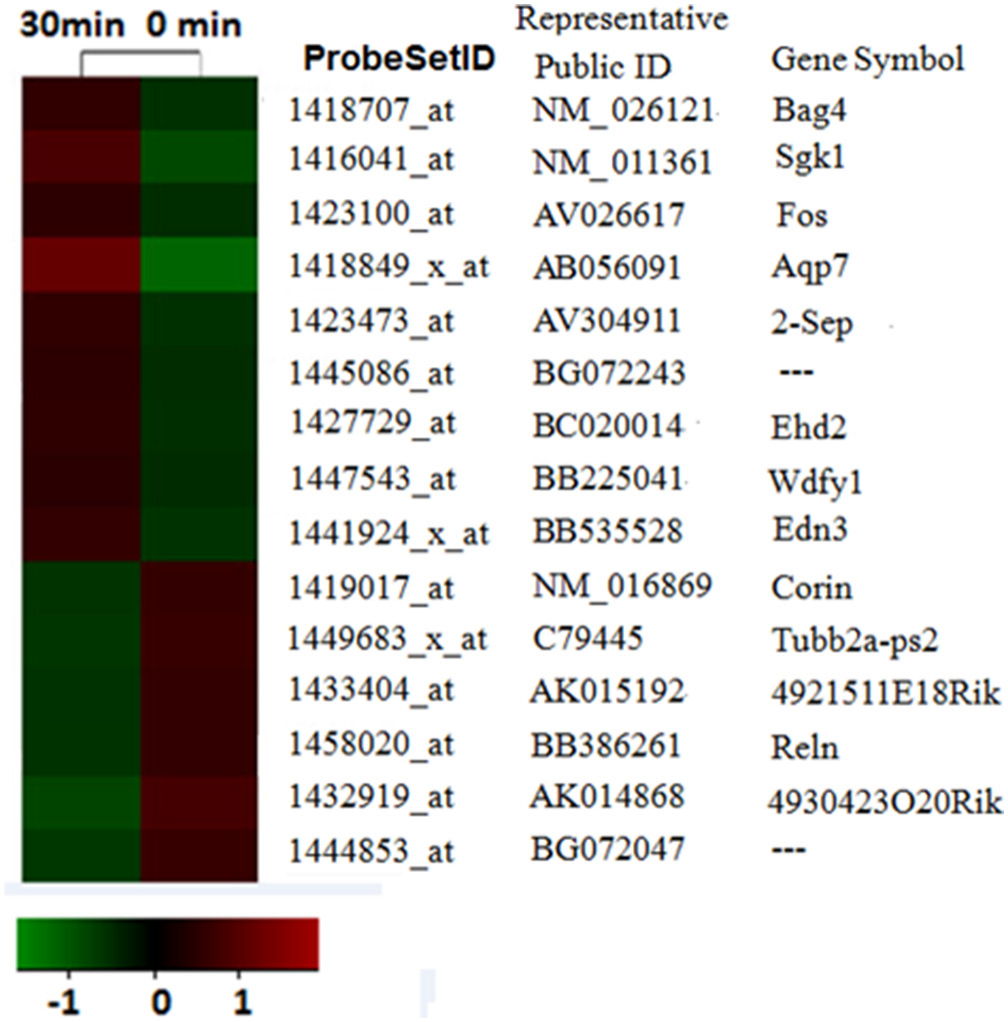
mRNA profiling analysis reveals itch-related altered genes The probe sets whose expressions were altered in compound 48/80 evoked-acute itching were identified by microarray analysis. The expression levels of each probe set (each line represents a single probe set) were displayed as a log2 ratio of their expression values divided by their expression value in sham mice.

### Real-time quantitative PCR (qPCR) validation of mRNA expression in compound 48/80-evoked pruritus mice

To validate the reliability of the microarray results, we analyzed these differentially expressed (DE) mRNAs, including 8 up-regulated mRNA and 4 down-regulated mRNA, by qPCR. The spinal cord (C_5_-C_8_) tissues were collected from control group (naive mice) and itch group (compound 48/80 treated mice). Six up-regulated mRNA, including Sgk1, Bag4, Fos, Ehd2, Edn3 and Wdfy were significantly increased, and three down-regulated mRNA, including Corin, 4921511E18Rik and 4930423020Rik were significantly decreased. qPCR results of three mRNA, including Aqp7, 2-sep and Reln were not consistent with data from microarray.

### The expression of 9 mRNA in the upper cervical spinal cord (C1-C4) from compound 48/80-evoked pruritus mice

**Figure 3:**
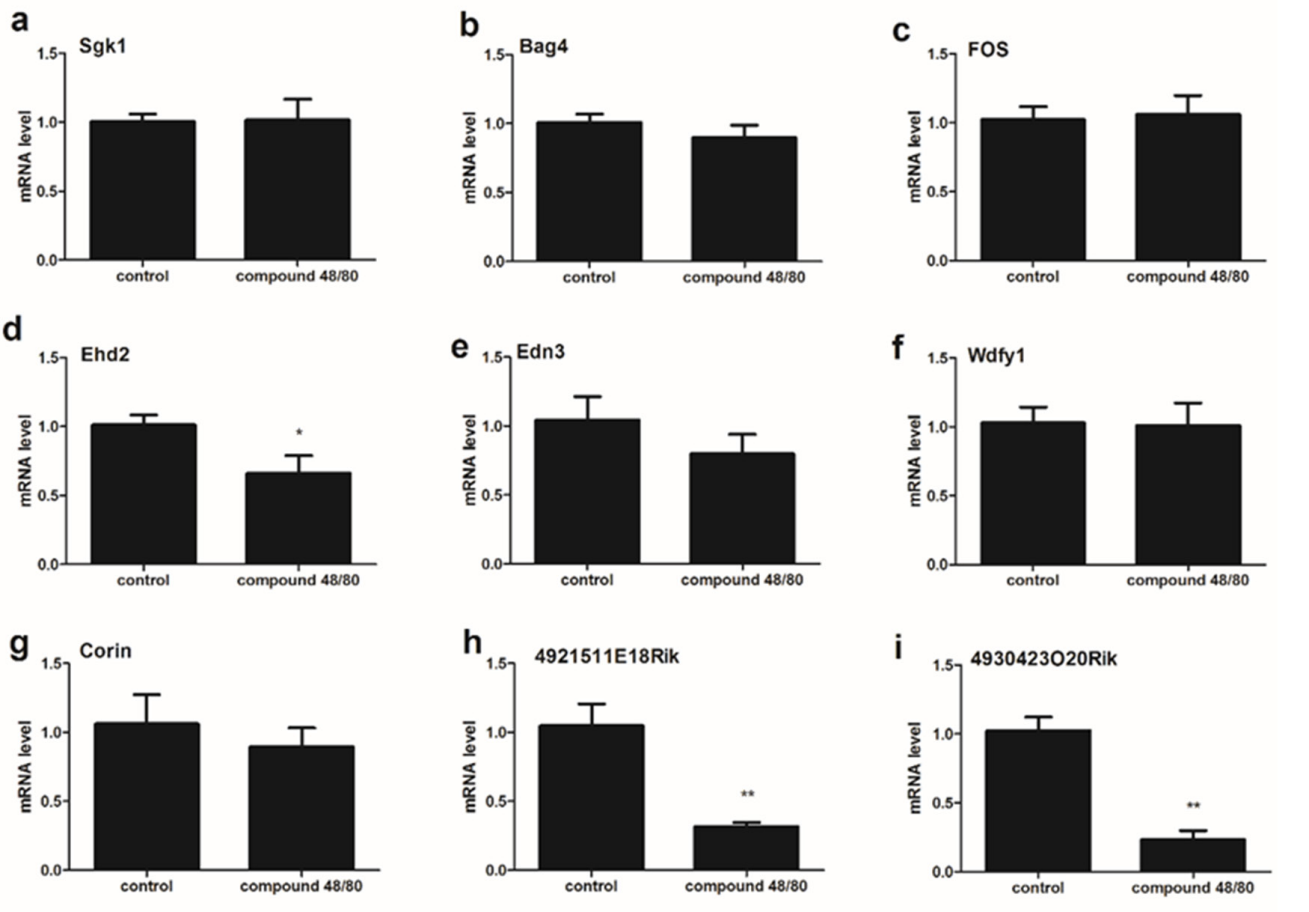
The expression of 9 mRNA in the spinal cord (C1-C4) from compound 48/80-evoked pruritus mice The expression of mRNA Ehd2 **(d)**, 4921511E18Rik **(h)** and 4930423O20Rik **(i)** was significantly down-regulated. The expression of 9 mRNA Sgk1 **(a)**, Bag4 **(b)**, Fos **(c)**, Edn3 **(e)**, Wdfy1 **(f)** and Corin **(g)** had no statistically different between control group and itch group. Mann-Whitney test.

To examine that if there is the differential expression of 9 mRNA between C1-C4 and C5-C8 in spinal cord, we validated the expression of 9 mRNA in the upper cervical spinal cord (C1-C4) from compound 48/80-evoked pruritus mice. Our results showed that the expression of mRNA Ehd2 (d), 4921511E18Rik (h) and 4930423O20Rik (i) was significantly down-regulated in 48/80 group (Figure 3), and the expression of 9 mRNA Sgk1 (a), Bag4 (b), Fos (c), Edn3 (e), Wdfy1 (f) and Corin (g) had no statistically different between control group and 48/80 group (Figure 3).

### The expression of 9 mRNA in the lumbar enlargement of spinal cord from compound 48/80-evoked pruritus mice

**Figure 4:**
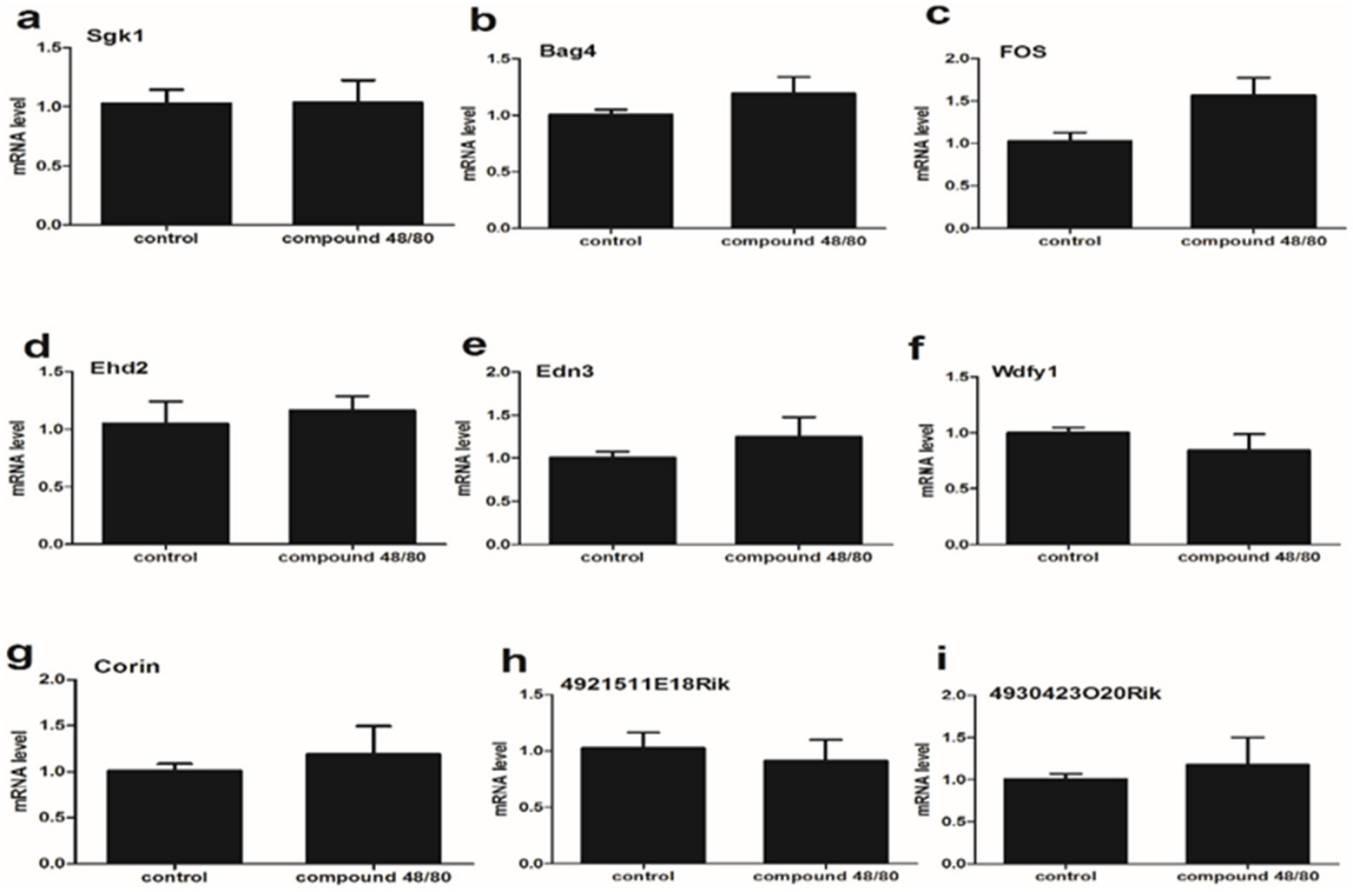
The expression of 9 mRNA in the spinal cord (lumbar enlargement) from compound 48/80-evoked pruritus mice **(a-i)** The expression of 9 mRNA had no statistically different between control group and itch group. Mann-Whitney test.

To examine if there is the differential expression of 9 mRNA between C5-C8 and the lumbar enlargement of spinal cord, we validated the expression of 9 mRNA in the lumbar enlargement of spinal cord (L3-L5) from compound 48/80-evoked pruritus mice. Our results showed that the expression of 9 mRNA had no statistically different between control group and itch group (Figure 4).

### The expression of 9 mRNAs in the spinal cord (C5-C8) at different time points (0.5h/2h) after compound 48/80 injection

**Figure 5:**
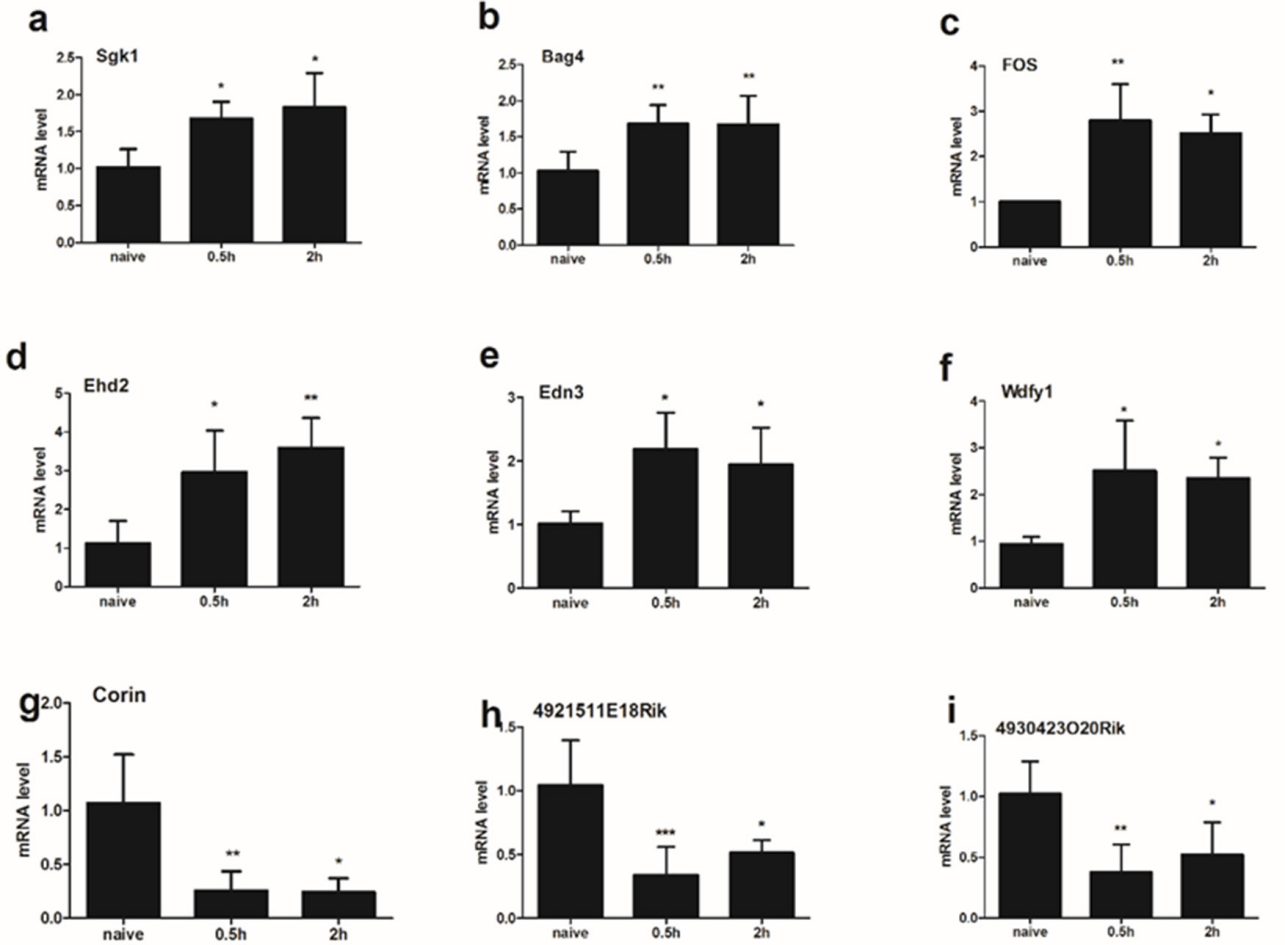
The expression of 9 mRNA were analyzed in the spinal cord (C5-C8) at different time points (0.5h/2h) after compound 48/80 injection The expressions of mRNA Sgk1 **(a)**, Bag4 **(b)**, Fos **(c)**, Ehd2 **(d)**, Edn3 **(e)**, and Wdfy1 **(f)** were significantly up-regulated underlying both 0.5h group and 2h group. The expression of mRNA Corin **(g)**, 4921511E18Rik **(h)** and 4930423O20Rik **(i)** was significantly down-regulated underlying both 0.5h group and 2h group. One-way ANOVA (Dunnelt: Compare all columns vs. naive column). *P < 0.05, **P < 0.01, ***P < 0.001.

Obviously, gene expressions after compound 48/80 injection are varied in different time points. We collected spinal tissue sample from C5-C8 0.5h vs 2h after compound 48/80 injection forRT-qPCR validation. Our results indicated that the expressions of mRNA Sgk1 (a), Bag4 (b), Fos (c), Ehd2 (d), Edn3 (e), Wdfy1 (f) and Reln (l) were significantly up-regulated underlying both 0.5h group and 2h group (Figure 5). The expression of mRNA Corin (g), 4921511E18Rik (h) and 4930423O20Rik (i) was significantly down-regulated underlying both 0.5h group and 2h group. One-way ANOVA (Dunnelt: Compare all columns vs. naive column) (Figure 5).

### RT-qPCR validation of mRNA expression in the spinal cord (C5-C8) from different itch models

**Figure 6:**
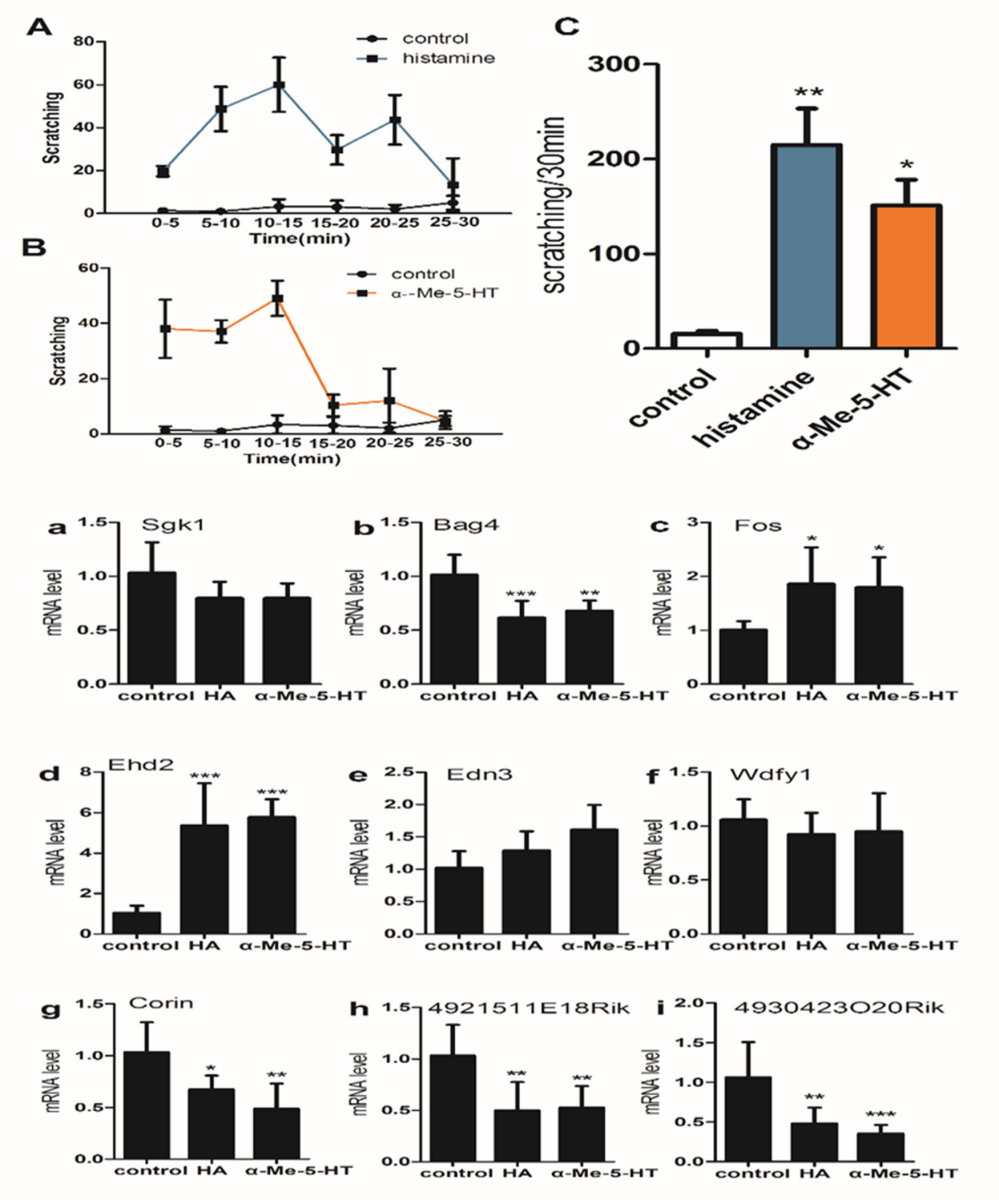
(upper) Pruritogen-evoked scratching behavior in histamine-treated (800μg/50μl), α-Me-5-HT-treated (35μg/50 μl) and saline-treated (control) mice (n = 6/group) **(C)** Graph by recording the total numbers of scratching bout at the 0-30min observation period by an intradermal microinjection of saline (15.67±5.13), histamine (215.00±66.57) and a-Me-5-HT (151.00±47.00). **(A, B)** Bar graph plots mean number of scratch bouts recorded at 5 min intervals. One-way ANOVA (Dunnelt: Compare all columns vs. control column). *P < 0.05, **P < 0.01. **(lower) The expressions of 9 mRNA were analyzed in histamine (HA)-treaded and α-Me-5-HT-treaded mice.** The expression of mRNA Fos and Ehd2 **(c, d)** were both significantly increased in HA group and α-Me-5-HT group. The expression of mRNA Bag4 **(b)**, Corin **(g)**, 4921511E18Rik **(h)** and 4930423O20Rik **(i)** were both significantly decreased in HA group and α-Me-5-HT group. The expression of mRNA Sgk1 **(a)**, Edn3 **(e)** and Wdfy1 **(f)** had no statistically different between control group and itch group. One-way ANOVA (Dunnelt). *P < 0.05, **P < 0.01, ***P < 0.001.

We further analyzed the expression of the 9 mRNAs which were in line with the array results in other two acute itch model including histamine (HA)-treated and α-Me-5-HT-treated mice (Figure 6). The expression of mRNA Fos and Ehd2 (Figure 6c, 6d) were significantly increased in HA group and α-Me-5-HT group. The expression of mRNA Bag4, Corin, 4921511E18Rik and 4930423O20Rik (Figure 6b, 6g, 6h, 6i) were significantly decreased in HA group and α-Me-5-HT group. The expression of mRNA Sgk1, Edn3 and Wdfy1 (Figure 6a, 6e, 6f) had not statistically different between control group and itch group.

### The expression of 9 mRNA in the spinal cord (C5-C8) from capsaicin-treated mice

**Figure 7:**
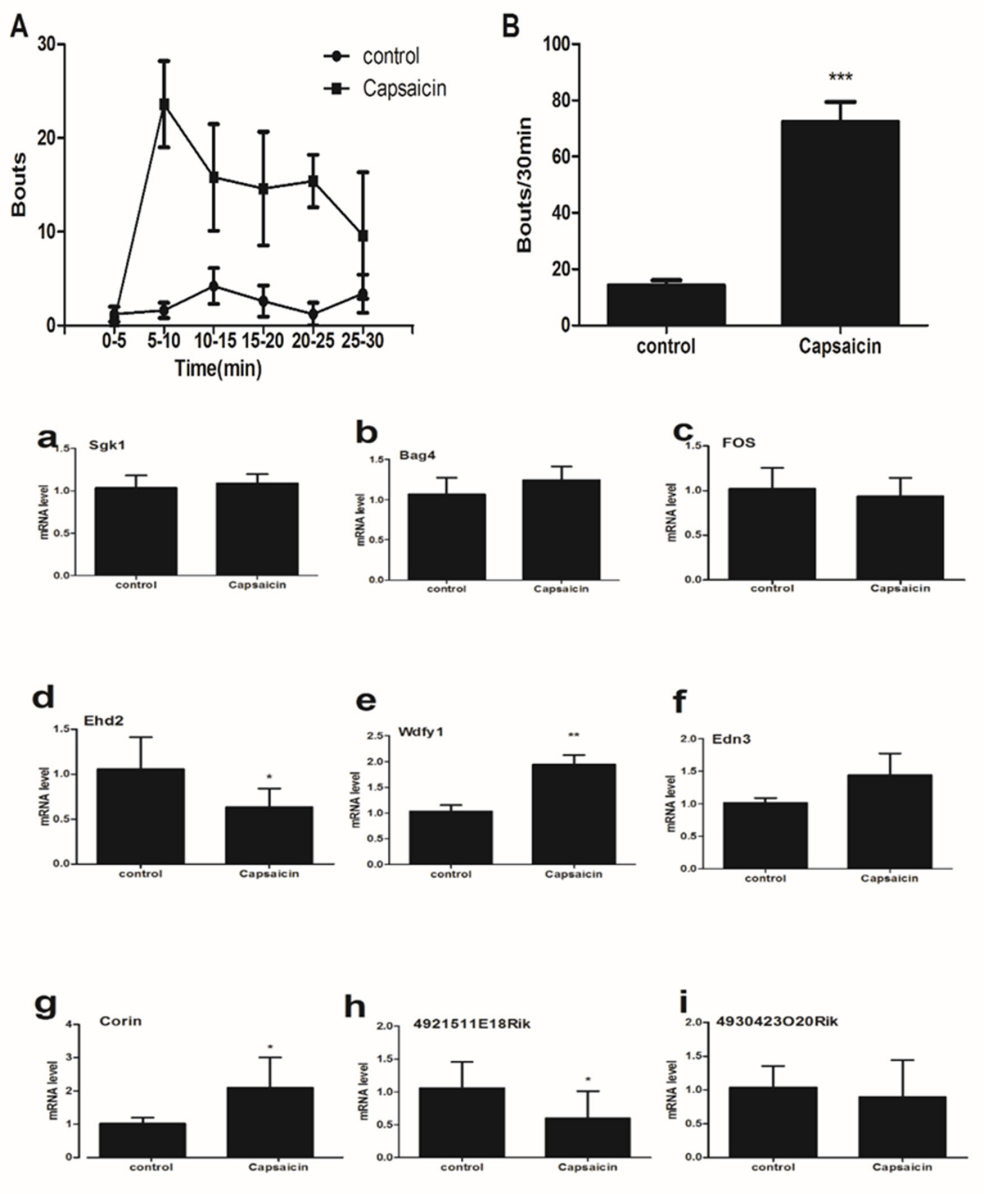
(upper) Bouts directed toward the site of injection of capsaicin (40μg/50 μl) into the nape of the neck **(A)**The time course shows bouts at 5-min intervals. **(B)** Mean total number of bouts during the 30 min period after injection of saline (15.67±5.13) and capsaicin (72.40±7.096). Unpaired t test. ***P < 0.001. **(lower) The expression of 9 mRNA in the spinal cord (C5-C8) from capsaicin-treated mice.** The expressions of mRNA Ehd2 **(d)**, Edn3 **(e)** and Corin **(g)** were significantly up-regulated in capsaicin group whereas the expression of mRNA 4921511E18Rik **(h)** was significantly down-regulated. The expression of mRNA Sgk1 **(a)**, Bag4 **(b)**, Fos **(c)**, Wdfy1 **(f)** and 4930423O20Rik (i) had no statistically different between control group and capsaicin group. Mann-Whitney test. *P < 0.05, **P < 0.01.

The time course of the wipes behaviors was compared between control group and capsaicin group. The total numbers of wipes -like behaviors during the 30-min test period was calculated in Figure 7. The difference between the capsaicin group and the control group was significant. RT-qPCR results that the expressions of mRNA Ehd2 (d), Edn3 (e) and Corin (g) were significantly up-regulated in capsaicin group whereas the expression of mRNA 4921511E18Rik (h) was significantly down-regulated, and the expression of mRNA Sgk1 (a), Bag4 (b), Fos (c), Wdfy1 (f) and 4930423O20Rik (i) hadn’t statistically different between control group and capsaicin group (Figure 7).

## DISCUSSION

Here, we identified that compound 48/80, histamine and α-Me-5-HT produced profound scratching behaviors, and these results were consistent with previous reports describing marked effects in mice responding to intradermal injection of a variety of pruritogens [18–26].

It is well-known that regulations of gene expression are varied in time course [27–30], and some gene could be regulated rapidly such as Fos, whereas some might be slow. 0.5h vs 2h after compound 48/80 injection, C5-C8 spinal tissue samples were collected forRT-qPCR validation. Our results indicated that there was no difference between 0.5h group and 2h group, and the expressions of 6 mRNA (Sgk1, Bag4, Fos, Ehd2, Edn3, Wdfy1 and Reln) were significantly up-regulated underlying 0.5h group and 2h group, whereas the expression of 3 mRNA (Corin, 4921511E18Rik and 4930423O20Rik) was significantly down-regulated. Further, some reports previously described that the change of scratching bout took place in the 0-0.5h observation period after pruritogen injection [6, 31]. Thus, in the present acute itching study, the spinal cord tissue samples of animals were detected 0.5 hour after the injection.

In the present report, we address if the intradermal injection of compound 48/80 induce a nonspecific response, since the blood absorption of compound 48/80 from the injection site may lead to the wide changes in gene expression in the whole spinal cord. Therefore, RT-qPCR validation between C1-C4 and lumbar spinal cord tissue was used to exclude the nonspecific response as a control. Our results showed that the expression of 9 mRNA (gk1, Bag4, Fos, Ehd2, Edn3, Wdfy, Corin, 4921511E18Rik and 4930423020Rik) in the spinal cord (lumbar enlargement) was no significant difference between control group and compound 48/80 group, suggesting that compound 48/80 injection don’t induce a nonspecific response by its blood absorption. Furthermore, the expression of 3 mRNA [Ehd2 (d), 4921511E18Rik (h) and 4930423O20Rik (i)] in the spinal cord (C1-C4) was significantly down-regulated, whereas the expression of 6 mRNA [Sgk1 (a), Bag4 (b), Fos (c), Edn3 (e), Wdfy1 (f) and Corin (g)] had no statistically different between control group and compound 48/80 group, suggesting that C1-C4 spinal cord segment may not be primarily involved in neuronal regulation of the injection site.

The spinal cord is a key hub for pruriceptive sensation and its signaling to the central nervous system (CNS) [32]. Pruritogen-triggered hyperexcitability in spinal cord is considered a major source of obstinate itching [33, 34]. The cellular hyperexcitability, in turn, is thought to be implicated in transcriptional switching in special cell body [16]. It is believed that analysis of microarray data of spinal cord may determine every mRNA transcript in a particular sample [35–39]. We selected 12 genes for RT-qPCR validation, and found that 75 % of the expression changes that were initially identified by the microarray data analysis were confirmed by RT-qPCR. These data revealed that the results from these microarray results had a high discovery rate. On comparing three itch models, we found that the various pruritogens yielded consistent results, and in three itch models, Fos and Ehd2 were up-regulated whereas Corin, 4921511E18Rik and 4930423020Rik were down-regulated. Furthermore, Corin and 4930423020Rik were down-regulated in itch model group compared to capsaicin group. A further study is needed in fact to address special function of these mRNA during acute itching.

In conclusion, the present results reveal that the application of microarray technique, coupled with RT-qPCR validation, further explain the mechanism behind itching evoked by pruritic compounds. Our data provides a powerful a useful baseline to begin functional experimentation involved in acute itching. It can thus contribute to our understanding of pharmacological methods for prevention or treatment of pruritus.

## MATERIALS AND METHODS

### Animal care

Male C57BL/6J mice (8-10 weeks old) were provided by the Experimental Animal Research Center of Hubei Province (license number: 42000600018764). The animals were maintained in a climate controlled room on a 12-hlight/dark cycle (light on at 07:00 h). Mice were housed (5/cage), but they were individually caged during each experiment. Food and water were available *ad libitum*. Experimental protocols were approved by the Institutional Animal Care and Use Committee of Tongji Hospital (No.TJ-A20150803). All testing and surgeries were performed in accordance with the policies and recommendations of the National Guides for the Care and Use of Laboratory Animals.

### Experimental design

**Experiment A** Mice were randomly assigned to two groups: (1) control group (saline 100μl, n = 18); (2) compound 48/80 group (50 μg/50μl, n = 18). After injection, pruritic behavior was immediately measured every 5 min. 0.5h after injection, cervical segment of spinal cord (C5-C8) tissue were prepared for mRNA profiling microarray analyses, and cervical (C1-C4, C5-C8) and lumbar segment of spinal cord for Real-Time quantitative PCR (RT-qPCR).

**Experiment B** Mice were divided into saline group (control group, *n*=12) and compound 48/80 group (48/80 group, *n*=12). 0.5h and 2h after injection, C5-C8 tissue was prepared for RT-qPCR.

**Experiment C** Mice were randomly assigned to three groups: (1) control group (saline 100μl, n = 6); (2) histamine group (800μg/50μl, n = 6); (3) α-Me-5-HT group (35 μg/50μl, n = 6). 0.5h after injection, C5-C8 tissue was prepared for RT-qPCR.

**Experiment D** Mice were randomly assigned to two groups: (1) control group (saline 100μl, n = 6); (2) capsaicin group (40μg/50μl, n = 6). Algogenic behavior was immediately measured every 5 min after injection. 0.5h after injection, C5-C8 tissue was prepared for RT-qPCR.

The protocols of each experiment were presented in the parts of figure legends. All procedures and behavioral assay were conducted in an isolated quiet room to reduce variance. An illustration of the experimental design above is displayed in Figure 1.

### Pruritic/wipes behavior

To induce acute itching on the skin, we followed a previously reported procedure [6, 31, 40–42]. Animals were received an intradermal microinjection of 100 μl saline, compound 48/80 (50μg/50μl), histamine (800μg/50μl), α-Me-5-HT group (35μg/50μl) or capsaicin (40μg/50μl) in the nape of the neck (via a 0.3 ml insulin syringe), respectively. Compound 48/80, histamine, α-Me-5-HT and capsaicin were purchased from Sigma-Aldrich (St. Louis, MO, USA). All drugs were dissolved in saline. All pruritic behaviors were evaluated as previously described with minor modifications [6, 43, 44]. The tests of algogenic behavior by capsaicin were performed similar with previous researches [11, 43].

### Tissue preparation

After pruritic behavior assay, the animals were immediately decapitated by cervical dislocation. Following decapitation, cervical (C1-C4, C5-C8) or lumbar segments in spinal cord were dissected using a dissection microscope and taken for subsequent analysis. The tissue was flash frozen in liquid nitrogen. Total RNA was isolated using Trizol^®^ reagent (Invitrogen, Carlsbad CA). RNA samples were performed by Ambion mirVana miRNA Isolation Kit for purity and concentration.

### mRNA profiling microarray analyses

High quality samples containing 200 ng of total RNA were used on microarray chips according to the manufacturer’s instructions. RNA was collected from six animals for each experimental condition. Gene profiling of C5-C8 spinal cord from control group and 48/80 group were carried out using standard Affymetrix protocols and hybridized to Affymetrix Mouse Genome 430 2.0 Array as described previously [45]. All subsequent analyses were conducted using SAM3.0 software (Stanford University, Stanford CA; http://www-stat.stanford.edu/^~^tibs/SAM).

### Real-time quantitative PCR

2 μg of total RNA was extracted from mouse spinal cords (C1-C4, C5-C8 or lumbar segment) using TRIzol reagent (Invitrogen, USA) according to the manufacturer’s protocol and quantified using a spectrophotometer (BioPhotometer, Eppendorf, Hamburg, Germany)[27, 46–49]. The threshold cycle (CT) was used to estimate the amount of target mRNA. The comparative CT method with the formula for relative fold-change = 2^-∆∆CT^was used to quantify the amplified transcripts. The specific forward (F) and reverse (R) primer sequences (Table 1) were designed [1]. Experiments were evaluated in triplicate.

**Table 1:** Primer sequences for RT-qPCR

Gene	Forward (5′ to 3′)	Reverse (5′ to 3′)
Sgk1	TGCCACCCTGGATCTATAACTG	GGCCTCAAAGTCTGACTCCC
Bag4	CCAAACACCTACCGTTCACCT	TGTGGTCGTCCAGTCCCTC
Aqp7	AAGGGCTTTCGTGCATCAGTA	ACTCCTATCCAGAAAACCGTCAA
Sept2	GGGTGGTGACAGTGACAGCG	CCTTCCTTCCACAGGGCTAA
Edn3	GCTTGCGTTGTACTTGTATGGG	GGTGGGCTTTATCTGTCCTTGA
Wdfy1	ATCAAGACCTATCCAGCCCACC	AAGCCCACGACGAGAAGAAG
Corin	AAAAGCGACCGAGATAAGAGTG	AAGCGCAGCAAGTTAGCAGT
Tubb2a-ps2	TGCATTTTGATGCCTTAGAAGT	CGCATGGTGCCTGGTTAG
Reln	CTTTGATGGCTTGCTGGTGA	GGTTGGTTGTAGGCAGGTGA
Fos	GCCCCTTCTCAACGACCC	CATCCCCAAGGAATTGCTGT
Ehd2	CGCAAGCTCAACGACCTAGT	TGAAGTCATGTGCCATCAACAG
4930423O20Rik	CTCAGCACCGACTCTTACACG	TGCTCTTGCTTCTTGCTCCTA
4921511E18	CGTCCCTGACCCCTACTCC	AAACTAGCAAGTGGCCCGTTA
β-actin	CACGATGGAGGGGCCGGACTCATC	TAAAGACCTCTATGCCAACACAGT

### Statistics and data analysis

Behavioral tests were performed by observers blinded to the treatments of the animals used. All quantification data are presented as mean ± SEM, and error bars represent SEM. Statistical comparisons were performed with Mann-Whitney test. The statistical analyses were done using t test, and P < 0.05 was considered statistically significant.
